# EXERCISE-BASED TELEREHABILITATION FOR PATIENTS WITH MULTIPLE SCLEROSIS USING PHYSICAL ACTIVITY: A SYSTEMATIC REVIEW

**DOI:** 10.2340/jrm.v56.40641

**Published:** 2024-11-13

**Authors:** Michaela SLADECKOVA, Jan KOCICA, Eva VLCKOVA, Filip DOSBABA, Garyfallia PEPERA, Jing Jing SU, Ladislav BATALIK

**Affiliations:** 1 Department of Neurology, University Hospital Brno, Brno; 2 Department of Rehabilitation, University Hospital Brno, Brno; 3 Department of Public Health, Faculty of Medicine, Masaryk University, Brno; 4 Faculty of Medicine, Masaryk University, Brno; 5 Department of Physiotherapy and Rehabilitation, Faculty of Medicine, Masaryk University, Brno; 6 Department of Rehabilitation, Faculty of Medicine, Masaryk University, Brno, Czech Republic; 7 Clinical Exercise Physiology and Rehabilitation Laboratory, Department of Physiotherapy, School of Health Sciences, University of Thessaly, Lamia, Greece; 8 School of Nursing, Tung Wah College, Hong Kong, China

**Keywords:** multiple sclerosis, telerehabilitation, rehabilitation, patient-reported outcome measures, exercise, systematic review

## Abstract

**Background:**

Telerehabilitation is a practical option for individuals with multiple sclerosis (MS) to engage in sustained physical activity without visiting a rehabilitation facility. The aim of this systematic review was to evaluate the feasibility, effectiveness, safety, and adherence of exercise-based telerehabilitation as compared with usual care for MS patients.

**Methods:**

A comprehensive literature search adhering to PRISMA guidelines was conducted, focusing on studies published in English since 2000. The systematic review protocol was registered in PROSPERO. The selection process involved strict criteria, including studies focusing on people with MS, telerehabilitation centred on regular exercise, a control group receiving usual care, valid exercise testing, and adherence to randomized controlled trial principles. Methodological quality was assessed using the TESTEX tool, ensuring rigour in study design and reporting.

**Results:**

Among the 281 records screened, 10 studies met the criteria. Telerehabilitation interventions varied in format and outcomes were assessed using diverse exercise tests and questionnaires. Despite variations, the studies collectively demonstrated promising feasibility and safety, with minimal withdrawals and minor adverse events. Effectiveness varied, with 5 out of 10 studies showing significant improvements in the intervention group. Adherence rates ranged from 38% to 100%.

**Conclusion:**

In most of the assessed aspects, telerehabilitation is comparable to regular centre-based rehabilitation.

## LAY ABSTRACT

This research explores a modern approach to helping people with multiple sclerosis (MS) stay active and improve their well-being through telerehabilitation. Our study reviewed 10 trials involving individuals with MS participating in telerehabilitation as compared with usual care. The findings suggest that telerehabilitation is a practical and safe option, with benefits similar to traditional rehabilitation. Although some studies demonstrated significant improvements, more research is needed to firmly establish the effectiveness of telerehabilitation. The study emphasizes the potential of telerehabilitation to provide accessible and effective support for people with MS, offering a flexible and convenient way to enhance their movement and overall quality of life.

Multiple sclerosis (MS) is an autoimmune disease that affects the myelin sheaths in the central nervous system. MS impacts over 2.8 million people globally, predominantly women of working age (69%) (1, 2). MS manifests variably with symptoms like vision loss, motor and balance disturbances, changes in sensory perception, and fatigue. Most patients (85%) initially experience the relapsing-remitting form, characterized by alternating attacks and periods of remission, which can significantly affect their psychological well-being, often leading to anxiety and depression (3, 4). As MS progresses, the patient’s independence and quality of life deteriorate. However, with a multidisciplinary approach it is possible to prevent disability and thus improve the patient’s quality of life.

Strong and consistent evidence suggests the health benefits of exercise-based rehabilitation in reducing morbidity and mortality in people with MS (5). Another benefit of regular moderate-intensity exercise in MS is the positive effect it has on certain cytokines that may play an important role in the development of an attack (6), as well as on cognitive functions such as memory and learning, which are also often affected in this disease (7). Although exercise is recommended, people with MS are less likely to engage in regular physical activity than the general population (8). Given the chronic nature of the disease, regular physical activity is essential. Centralized rehabilitation services can promote physical activity and exercise, but uptake is low. Alternative delivery models, such as telerehabilitation and mobile health, are recommended to reduce barriers to rehabilitation.

Telerehabilitation is one of the practical alternatives for providing patients with regular, long-term physical activity in their everyday lives without the need to visit a rehabilitation facility (9). The advantage from the patient’s perspective may be the independence of choosing where and when to exercise. In addition, telerehabilitation has the potential to reduce the costs to the healthcare system (10). It can be offered to a larger number of patients simultaneously and over a very long period. Nowadays, with the development of information and communication technology and the increasing availability of internet access, there is further scope for the development and implementation of telerehabilitation approaches in healthcare practice (11).

A critical aspect of telerehabilitation’s success lies in patient engagement, which is significantly enhanced by the use of both synchronous and asynchronous application methods (12, 13). Synchronous methods, such as live video consultations, enable real-time interaction between patients and healthcare providers, allowing for immediate feedback, correction, and motivation during rehabilitation exercises. On the other hand, asynchronous methods, including pre-recorded instructional videos, mobile apps, and digital platforms, allow patients to perform exercises at their own convenience, providing flexibility and autonomy in managing their rehabilitation schedules (14). The combination of these approaches can cater to different patient needs and preferences, thereby improving adherence to rehabilitation protocols (15). Additionally, advancements in wearable devices and sensors integrated into these methods enable continuous monitoring of patient progress, offering valuable data that healthcare providers can use to tailor interventions more precisely (16). This blend of synchronous and asynchronous methods not only enhances the effectiveness of telerehabilitation but also empowers patients by giving them a more active role in their recovery process.

In 2018, an extensive review focused on telemedicine for people with MS, considering telerehabilitation as well as usual care and mental health and neuropsychological care (17). The results of the study showed that telemedicine in a long-term intervention is beneficial and cost-effective for both patients and care providers (10). However, a more detailed focus on the description of telemedicine-based exercise interventions and tools to evaluate the effects of rehabilitation therapy is lacking. Due to the lack of trials, it remains unknown whether telerehabilitation is effective and safe, what the level of adherence to prescribed rehabilitation is, whether there are differences in the effectiveness of selected rehabilitation methods, and what the appropriate outcome measures are for testing patients (10, 11). Therefore, we aimed to review the literature on telerehabilitation in people with MS and assess the feasibility, effectiveness, safety, and level of adherence in telerehabilitation and determine whether any of the telerehabilitation methods appear to be more beneficial than usual care.

## METHODS

A comprehensive literature search was conducted to determine the impact of exercise-based telerehabilitation and compare it with usual care for people with MS. The systematic review was conducted in accordance with the Preferred Reporting Items for Systematic Reviews and Meta-Analyses (PRISMA 2020) guidelines (18). The review protocol was registered in the Prospective Register of Systematic Reviews (PROSPERO) registry (CRD42021277467).

### Search strategy

An electronic literature search was conducted in August 2024 using the PubMed database and the Web of Science metasearch engine. The search was structured to identify the effect of exercise-based telerehabilitation interventions published in English since 2000. Articles were selected from the Medical Subject Heading (MeSH) database that contained the following search terms: Multiple Sclerosis AND telerehabilitation OR mHealth OR internet OR mobile OR smart OR tele AND exercise therapy OR physical activity OR exercise OR training OR physical fitness OR rehabilitation. Two authors (MS, LB) independently carried out the initial selection process using titles and abstracts, from which studies with the potential to meet the inclusion criteria were selected. If the 2 authors reached different conclusions, they discussed the discrepancies and, if necessary, consulted a third researcher (FD) to reach a consensus and ensure a validated final selection. In addition, the authors of the study selection process hand searched the references of recent systematic reviews to identify any relevant studies that had not been revealed in the search.

### Inclusion criteria

- Studies focusing on patients with multiple sclerosis.
- Use of telerehabilitation focusing on regular exercise or physical activity.
- A control group of people with MS receiving usual care.
- Testing with a valid exercise test.
- Randomized controlled trial in English.

### Exclusion criteria

Trials were excluded if they used a health education approach to improve physical activity, if patients in the control group received active control treatment, if they were pilot studies (19), or if the full text was not available after contacting the authors.

### Evaluation of studies

The final selected studies were assessed for methodology, outcome assessment, bias, and validity using the TESTEX tool (20), as this tool is suitable for studies dealing with exercise interventions. TESTEX was developed exclusively to assess of the quality of exercise trials. TESTEX covers 12 criteria. A total of 15 points can be awarded, 5 points for study quality and 10 points for reporting. Depending on the number of points obtained, the study is classified as high quality (≥12 points), good quality (7 to 11 points), or low quality (≤6 points). Study quality is assessed in terms of inclusion and exclusion criteria, randomization, how patients were assigned to each group, the similarity of the groups, and the blinding of the investigator. Study reporting is assessed by outcome measures, whether the intervention is relevant to the study of interest, statistical comparisons between groups, reporting of *p*-values for each outcome, reporting of exercise levels for patients in the control group, gradual increase in exercise to achieve consistent patient load, and exercise characteristics (intensity, frequency, duration of intervention).

## RESULTS

**Fig. 1:**
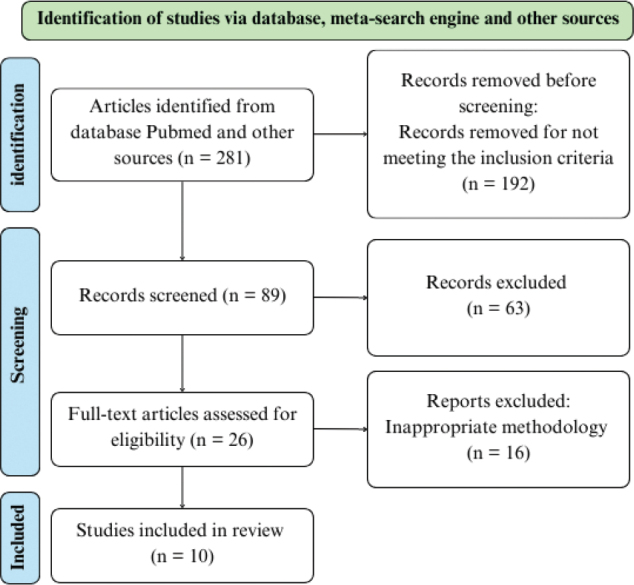
Flowchart detailing the search strategy (18).

A search of databases and meta-search engines was performed and 281 records were identified. After screening the titles and abstracts, 192 publications were found not to meet the inclusion criteria. The reasons for exclusion were that the trials were not randomized control trials or that they had an inconvenient design, an intervention, or a language other than English. After further screening, 63 studies were excluded due to the lack of a control group or the lack of a valid exercise test. After full-text screenings, 16 more trials were excluded due to inappropriate methodology or intervention. Finally, 10 publications met the inclusion criteria for this systematic review and meta-analysis. Fig. 1 provides an overview of the study process implemented according to PRISMA 2020 (18).

### Characteristics of the studies

Table I shows the selected studies and their design and exercise characteristics, including frequency, primary and secondary outcomes, sample size, and control group characteristics. All selected trials were randomized controlled trials. Three studies were from the United States (24, 29, 31), 2 were from Germany (26, 28) and 1 each were from Australia (25), the United Kingdom (27), Spain (30) Italy (32), and Turkey (33). Five studies were less than 5 years old (25, 28, 29, 32, 33) and 5 were published in 2014 (24, 26, 27, 30, 31). The number of subjects in the experimental groups ranged from 10 to 139, and the total number of subjects in the experimental groups was 326. The duration of the intervention ranged from 6 weeks to 48 weeks. The frequency of exercise, which ranged from 2 days per week to daily exercise, was individually determined by the participant. In 5 cases, the control group received usual care (24, 27–30); in 1 case, centre-based rehabilitation was provided (25); and in 4 cases, the control group received usual care for the duration of the trial and was offered an intervention after the experimental period (26, 31–33). Five studies used internet platforms (24, 27, 28, 32, 33) or telephone/email follow-up (26, 28, 29) and 3 online diaries (25, 30, 31). The form of telerehabilitation varied. Most studies used conventional exercises and focused on lower limb strength and endurance, with only 1 trial focusing on upper limb strength and endurance (30). One study provided Pilates, which focuses on increasing the strength and endurance of the whole body (33). In some studies, the participant received an educational seminar in which the principles of the exercises were taught (26, 30, 31, 33). In other studies, the participant had an individual lecture in which the physiotherapist chose individually tailored exercises (24, 25, 27–29). One trial used 2 weeks of supervised training (32).

**Table I:** Overview of studies, including design, interventions and group characteristics

Author (publication year and country)	Type of intervention	Duration (exercise frequency)	Main outcome measures	Secondary outcome measures	IG: number of subjects	CG: number of subjects (intervention)	EDSS: type of MS inclusion criteria	Mean age (IG/CG)	Type of MS, *n* Benign/RRMS/PPMS/SPMS/NR	Mean EDSS
Conroy et al. (2017, USA) (24)	MS HAT – platform for data collection, educational content, exercise information and therapist–patient communication	48 weeks (daily)	T25FW	6MWT, BBS, MSWS-12	16	8 (common handout for PT home exercise)	NR, RRMS, SPMS, PPMS	50.4/53.3	0/8/1/15/0	NR
Williams et al. (2020, Australia) (25)	Exercises for improving gait, telephone support every two weeks	8 weeks (2x60 min per week)	10mWT	6MWT, BBS	24	26 (centre-based rehabilitation)	NR, RRMS, SPMS, PPMS, benign	51.3/52.7	3/31/7/6/3	NR
Tallner et al. (2016, Germany) (26)	E-training – platform for delivering exercises in PDF documents and communication with therapist	48 weeks (number of training units was individual)	HRQoL	Muscle strength, aerobic capacity, lung function, physical activity, fatigue	36	41 (waiting list)	≤4.0, RRMS, SPMS	40.9/40.7	0/52/0/7/0	2.7
Paul et al. (2014, UK) (27)	Web-based physiotherapy – website with a video, text explaining and an audio description of exercises	12 weeks (2x per week)	T25FW	BBS, TUG, MSIS-29, Leeds QoL, MS-related symptom checklist, HADS	15	14 (usual care)	5.0–6.5, RRMS, SPMS, PPMS	50.8/52.5	2/17/4/5/2	5.9
Flachenecker et al. (2020, Germany) (28)	Internet-based physical activity promotion programmes	12 weeks (1-2x per week)	WEIMuS	MSIS-29, gait 2 min/ 10 m, Tinetti score	34	30 (usual care)	≤6.0, RRMS, SPMS, PPMS	47.6/46.4	NR/49/NR/NR/NR	4.2
Plow et al. (2019, USA) (29)	Individually tailored phone calls Telephone-delivered interventions	14 weeks	FIS, GLTEQ	MSIS-29, moderate-to-vigorous exercise, accelerometer step count	69 PA/70 PA + fatigue management	69 (usual care)	NR, RRMS, SPMS, PPMS	51.2/51.2/51.8	0/176/7/11/14	NR
Ortiz-Rubio et al. (2016, Spain) (30)	Supervised, individually developed home-based upper limb training	8 weeks (2x60 min per week)	FTT, ARAT	PPT	19	18 (booklet with exercises)	<7.5 RRMS	42.2/44.9	0/8/5/24/0	5.9
Sosnoff et al. (2014, USA) (31)	Home-based exercise programme focussed on balance, lower limb muscle strength, core muscle strength and stretching	12 weeks (3x per week)	Physiological Profile Assessment – fall risk score	Mobility and balance outcomes, self-reported falls, T25FW, 6MWT, TUG, MSWS-12, BBS, ABC	10	12 (waiting list)	2.5–6.5 RRMS, SPMS, PPMS	60.1/60.1	0/20/3/4/0	5.0
Straudi et al. (2022, Italy) (22)	10 supervised task-oriented circuit training sessions (2 weeks) followed by a 12-week home-based task-oriented programme	14 weeks (3x60 min per week)	6MWT	T25FW, TUG, MSWS-12, MFIS, DGI, MSIS-29, rmVO2	18	18 (waiting list)	4.0–5.5 RRMS SPMS PPMS	49.6/52.6	0/15/11/10/0	4.67
Eldemir et al. (2024, Turkey) (33)	Pilates based telerehabilitation via video call	6 weeks (3x60 min per week)	Muscle strength (shoulders, hips, knees, ankles)	Core endurance and power, BBS, gait analysis, 6MWT, FSS, FIS, MSQOL-54	15	15	0.0–5.0 NR	41/38.4	NR	1.5

ABC: Activities-specific Balance Confidence scale; ARAT: Action Research Arm Test; BBS: Berg Balance Scale; CG: control group; DGI: Dynamic Gait Index; EDSS: Expanded Disability Status Scale; FIS: Fatigue Impact Scale; FSS: Fatigue Severity Scale; FTT: Finger Tapping Test; GLTEQ: Godin Leisure-Time Exercise Questionnaire; HADS: Hospital Anxiety and Depression Scale; HRQoL: health related quality of life; IG: intervention group; LEEDS QoL: Leeds Quality of Life scale; MFIS: Modified Fatigue Impact Scale; MS: multiple sclerosis; MS HAT: Multiple Sclerosis Home Automated Tele-management system; MSIS-29: Multiple Sclerosis Impact Scale; MSQOL-54: Multiple Sclerosis Quality of Life-54; MSWS-12: Twelve Item MS Walking Scale; NR: not reported; PA: physical activity; PPMS: primary progressive multiple sclerosis; PPT: Purdue Pegboard Test; PT: physical therapy; rmVO2: resting muscle oxygen consumption; RRMS: relapsing-remitting multiple sclerosis; SPMS: secondary progressive multiple sclerosis; TUG: Timed Up and Go test; T25FW: Timed 25-Foot Walk test; WEIMuS: Würzburger Erschöpfunsinventar bei Multipler Sklerose; 6MWT: Six-Minute Walk Test; 10mWT: Ten Meter Walk Test.

A total of 632 people participated, 19 different exercise tests were examined, 13 different questionnaires were used, and in 1 study additional values were obtained from routine physiological measurements (21). The exercise tests focused on gait, balance, and upper limb function; these tests complement the commonly used Expanded Disability Status Scale (EDSS) to report clinical outcome measures (COMs) (21, 22). Patient-reported outcome measures (PROMs) were obtained using questionnaires to add the patient’s perspective to the clinical data (23). The questionnaires focused on gait (24, 31, 32), quality of life (26–29, 32, 33), fatigue (26, 28, 29, 32, 33), depression (27), disability (27), and frequency of physical activity (27, 29).

### Evaluation of individual methodologies

Studies evaluating the effect of telerehabilitation on movement skills vary widely in the primary outcomes chosen. Five studies (24, 25, 28, 30, 32) used movement skills tests as the primary outcome, and 1 study assessed muscle strength in the limbs for this purpose (26). Other studies used a questionnaire survey for the primary outcome (26, 28, 29, 31). Of the primary movement outcomes, 4 studies focused on movement skills related to lower extremity function (24, 25, 27, 32), 1 on upper extremity function (30) and 1 on both (33). Two studies measured the timed 25-foot walk (T25FW), a test that measures how long it takes a patient to walk a distance of 25 feet (34). One study used the 10-metre walk test (10mWT), similar to the T25FW. One study used the 6-minute walk test (6MWT) to measure endurance walking: the patient walks back and forth on a 25-m track for 6 min and the distance walked in that time is measured. Two tests were selected for upper extremity testing, the Finger Tapping Test (FTT), in which the tester measures the speed of the index finger tapping on a table for 10 s on both sides (35), and the Action Research Arm Test (ARAT), which assesses upper extremity function using 19 tasks (36). The 6MWT (24, 25, 31, 33), T25FWT (31, 32), and 10mWT (28) were selected as secondary movement outcomes.

In addition, 5 studies used the Berg Balance Scale (BBS) to assess balance (24, 25, 27, 31, 33). This scale assesses the ability to perform fourteen balance tasks safely (37). Tests to assess balance include the Timed Up and Go Test (TUG) (38), in which the patient is asked to stand up from a chair without using their arms, walk 3 m, turn around, return, and sit back down in the chair. This test was used in 3 trials (27, 31, 32). A 2-minute walk test (2MWT) was used in 1 study (28). Three trials measured locomotor skills with non-specific tests, using dynamometer-measured muscle strength, core endurance, and power, and accelerometer-measured steps taken during the day (26, 29, 33). Table II shows the results of selected exercise tests and their comparison before and after the intervention.

**Table II:** Between-study statistical intervention overview and safety

Author, publication year	Type of primary outcome	Experimental group statistics	Control group statistics	Between-group analysis	Study adherence	Adverse events
Conroy et al. 2017 (24)	T25FW	*p* = 0.28	*p* = 0.38	*p* = 0.44	50%	Not specified
Williams et al. 2020 (25)	10mWT	Minimal clinically important differences were reported	Minimal clinically important differences were reported	Minimal clinically important differences were reported	45%	None
Tallner et al. 2016 (26)	Muscle Strength (KE/KF; TE/TF); sports activity	*p* = 0.003/ < 0.001; 0.8/0.001; < 0.001	*p* = 0.81/0.3; 0.99/0.02; 0.39	*p* = 0.02/0.003; 0.85/0.35; 0.001	36%	Not specified
Paul et al. 2014 (27)	T25FW	Effect size Cohen’s d = 0.44	Effect size d = 0	*p* = 0.17	average logins from 2.1 to 0.9	3 unrelated adverse events
Flachenecker et al. 2020 (28)	10mWT/2 MWT	*p* < 0.01/*p* < 0.01	*p* < 0.02/*p* < 0.01	Not specified	65%	Not specified
Plow et al. 2019 (29)	Step count accelerometers	Not specified	Not specified	PA only vs. CG p < 0.01	61%	Not specified
Ortiz-Rubio et al. 2016 (30)	FTT M / FTT L; ARAT M / ARAT L	*p* = 0.061/ 0.003; 0.041/0.038	*p* = 0.407/0.145; 0.454/0.187	*p* = 0.004/0.064; < 0.001/< 0.001	100%	None
Sosnoff et al. 2014 (31)	T25FW	21.7% acceleration	3.1% acceleration	*p* = 0.04	68%	2 unrelated adverse events
Straudi et al. 2022 (32)	6MWT	*p* < 0.001	*p* = 0.12	*p* < 0.001	62%	Not specified
Eldemir et al. 2024 (33)	Muscle strength (SFR/SFL; SAR/SAL; HFR/HFL; HER/HEL; HAR/HAL; KFR/KFL; KER/KEL; ADR/ADL)	< 0.001/0.001; < 0.001/0.998; < 0.001/< 0.001; 0.001/0.001; 0.005/0.51; 0.033/0.002; 0.006/0.001; 0,02/0.005; 0.057/< 0.001	No significant changes	Not specified	97.3%	None

ADL: ankle dorsiflexion left; ADR: ankle dorsiflexion right; ARAT L: Action Research Arm Test less affected upper limb; ARAT M: Action Research Arm Test more affected upper limb; CG: control group; EDSS: Expanded Disability Status Scale; FTT L: Finger Tapping Test less affected upper limb; FTT M: Finger Tapping Test more affected upper limb; FM: fatigue management; HAL: hip abduction left; HAR: hip abduction right; HEL: hip extension left; HER: hip extension right; HFL: hip flexion left; HFR: hip flexion right; IG: intervention group; KE: knee extension; KEL: knee extension left; KER: knee extension right; KF: knee flexion; KFL: knee flexion left; KFR: knee flexion right; m: months; MS HAT: Multiple Sclerosis Home Automated Tele-management system; PA: physical activity; SAL: shoulder abduction left; SAR: shoulder abduction right; SFL: shoulder flexion left; SFR: shoulder flexion right; TE: trunk extension; TF: trunk flexion; T25FW: Timed 25-Foot Walk test; 6MWT: Six-Minute Walk Test; 10mWT: Ten Meter Walk Test.

### Feasibility

The studies do not mention any difficulties with the feasibility of telerehabilitation in the home environment. Only in 1 case did the participant withdraw from the study due to technical problems (28). Other reasons for dropping out (loss of motivation, stress, lack of time) were not directly related to telerehabilitation. Often participants did not give a specific reason. However, some studies mentioned that there is a need for further research into not only the appropriate type of telerehabilitation, but also the appropriate way to motivate patients for long-term adherence (25, 26). Nevertheless, there was a general agreement that telerehabilitation could be an appropriate, personalized, holistic, accessible, and cost-effective rehabilitation option for people with MS (24–33).

### Effectiveness

Five out of 10 studies showed a significant improvement in the intervention group (26, 28, 30, 32, 33). Six studies reported a significant difference between the intervention group and the control group (26, 28–32). Only 2 studies (26, 28) also showed improvement in the control group; both those trials took place in Germany. No other German studies were included because no other studies met the inclusion criteria.. The trials showing effectiveness in the intervention group had different intervention durations, ranging from 6 weeks to 48 weeks. Therefore, we include the longest and shortest interventions in these trials (29, 33). The study approaches to telerehabilitation varied, and a lack of data makes it impossible to determine the ideal length of intervention and type of telerehabilitation for effective rehabilitation.

### Study quality

The quality of the studies was measured using the TESTEX tool (20); the selected studies were of good overall quality with a mean score of 10.4 (Table III). The mean study quality score was 4.5 points (range 3–5 points), which is considered high quality, and the mean study reporting score was 5.9 points (range 3–8 points), which is good quality.

**Table III:** The TESTEX study quality evaluation

Author, publication year	Eligibility criteria	Randomi-zation specified	Allocation conceal-ment	Similar groups at baseline	Blinding of a assessor	Outcome measures	Intention-to-treat analysis	Between-group statistical	Point of variability measures*	Activity monitoring in CG	Relative exercise intensity	Exercise volume and energy	Summary
Conroy et al. 2017 (24)	1	0	0	1	1	1	1	2	1	1	1	0	10
Williams et al. 2020 (25)	1	1	1	1	1	2	1	2	1	1	1	0	12
Tallner et al. 2016 (26)	1	1	1	1	1	1	1	1	0	0	1	0	11
Paul et al. 2014 (27)	1	1	0	1	0	2	1	0	0	0	1	0	8
Flachenecker et al. 2020 (28)	1	1	1	1	1	2	1	1	0	0	1	0	11
Plow et al. 2019 (29)	1	1	1	1	1	1	1	0	0	0	0	0	8
Ortiz-Rubio et al. 2016 (30)	1	1	1	1	1	3	1	1	1	1	0	0	13
Sosnoff et al. 2014 (31)	0	1	1	1	1	2	1	1	0	0	1	0	11
Steraudi et al. 2022 (32)	1	1	1	1	1	1	1	1	0	0	0	0	10
Eldemir et al. 2024 (33)	1	1	1	1	1	3	1	0	0	0	1	0	10
Mean													10.4

* Point measures and measures of variability for all reported outcome measures.

CG: control group.

### Adherence

Two studies of 6–8 week duration showed adherence of over 95% (30, 33). Five trials (lasting 12 to 14 weeks) showed an adherence rate of 67% ± 6% (26, 28, 29, 31, 32). In 1 trial, patients were followed for a further 12 weeks after the initial 12 week trial, and adherence dropped from 73% to 36% (26). In a study where rehabilitation was conducted both at the centre and at home under identical conditions for 8 weeks, adherence to home-based exercises was 38% lower than adherence to centre-based exercises (45% adherence at home vs 83% at the centre) (25). The longest study (48 weeks) reported an adherence rate of 50% (24).

### Safety, incidence of adverse events (AE)

Five trials described adverse events (AEs) (25, 27, 30, 31, 33). Three studies did not report the occurrence of AEs (25, 30, 33). One study reported 3 AEs: elbow fracture, hospitalization for infection, and breast cancer diagnosis (27). In another study, a fall resulted in the fracture of a bone in the foot and a worsening of the disease (30). One fracture and 1 exacerbation of the disease occurred in patients in the experimental group; the other adverse events were reported in the control groups. The adverse events that did occur were reported to be unrelated to the exercise intervention in both trials (27, 31).

## DISCUSSION

### Feasibility

Advances in information and communication technology in recent years, combined with limited opportunities for face-to-face contact during the COVID-19 pandemic, have led to the emergence of telemedicine and associated telerehabilitation. The use of digital options can reduce the length of hospital stay (39), and another study suggested that this approach was cost-beneficial (40). Telerehabilitation could be defined as a set of tools that enable the patient to perform rehabilitation effectively and safely at a remote location (41), and telerehabilitation programmes can be as feasible and effective as conventional physiotherapy (42). Telerehabilitation can be delivered through audio/video telephone calls, web platforms with specific software, text messages, or emails, and sensors that record selected patient parameters (heart rate, number of steps) that can be reviewed by medical staff virtually asynchronously or in real time. A systematic review by Seron et al. (2021) (43) showed the low quality of telerehabilitation research in physiotherapy. Despite this, 3 studies showed the feasibility and similar effect of telerehabilitation compared with usual care in people with MS. This is consistent with our findings: none of the trials showed a deterioration in the experimental group compared with the usual care control group; on the contrary, 6 trials showed a significant improvement (26, 29–33).

### Adherence

The guideline-based MS project by Learmonth et al. (44) highlighted in 137 patients with MS that home-based exercise training has significant benefits, and a large proportion of patients (71%) adhere to all exercise sessions. This is in line with our results; we saw varying levels of adherence, with the average adherence for the 12-week intervention being around 67%. A meta-analysis by Dennett et al. (45) reported that adherence rates were relatively consistent, with 41 exercise interventions for people with MS showing an average adherence rate of 73%. This emphasizes the need for home-based exercise programmes that can be easily implemented and adhered to by people with MS. Motl et al. (46) recommended that future research should focus on optimizing adherence and compliance to maximise the benefits of exercise training.

### Effectiveness

Several studies have investigated the effectiveness of home-based exercise programmes for people with MS. Home exercise and physiotherapy programmes have been shown to be significantly more effective than a control group intervention for managing depression (medium effect) and for quality of life (low effect) in people with MS (47, 48). In addition, Ghahfarrokhi et al. (49) found that home exercise programmes were effective in improving a range of outcomes, including walking ability, balance, muscle strength, and fatigue.

The home-based telerehabilitation interventions in this review reported different outcomes, making data synthesis challenging. Some similarities in the review can be seen in the studies by Tallner et al. (26) and Eldemir et al. (33), where muscle strength was chosen as the primary outcome measure, and both studies have significant findings. This suggests that muscle strength can be a suitable outcome measure, but more studies using this outcome measure are needed. Another common sign is that 7 trials used an activity that improved both strength and endurance (24–28, 32, 33). In general, however, they suggest a significant effect on motor skills compared with the control group. The types of these interventions that appear to be effective were studied in patients with moderate (26, 32) to severe (28, 30) disability, and this showed that home exercise supported by telerehabilitation can have a positive impact on physical function and symptom management. Overall, the available evidence suggests that home-based exercise programmes are effective in improving a variety of outcomes, including physical functioning, quality of life, cognition, balance, and fall risk in people with MS (50). These programmes offer patients a convenient and affordable way to exercise regularly and manage their symptoms. At the same time, the studies in the review met the recommended amount of physical activity per week according to guidelines by Kim et al. (2019) for general resistance exercise prescription (51). However, larger cohorts and clarification of the appropriate exercise protocol are needed.

### Quality of studies

In 2015, Khan et al. (52) demonstrated the low methodological quality of telerehabilitation studies in their systematic review. A more recent systematic review assessed the quality of 8 studies as average and 3 as good (53). In our review of the quality assessment of the studies, the standard of design and outcome reporting was good despite the methodological diversity of interventions and outcomes. Only 2 of the 10 studies were below the mean, suggesting that this collection of studies is a good sample with a strong and reproducible research base. This finding shows progress in this area of research, as the quality of telerehabilitation studies in MS has improved since the last review (50, 53). However, many different exercise tests or questionnaires were used across the sample, highlighting the need to establish basic recommendations for appropriate tools and outcomes to evaluate the impact of home-based telerehabilitation interventions in MS.

### Safety

Regular telemonitoring and telecounselling by health professionals is essential to ensure safety, correct dosage, and exercise technique. It is important that emerging trials in this area report on the occurrence of AEs, as only 5 trials in this systematic review addressed the safety of exercise (25, 27, 30, 32, 33). In 3 trials (25, 30, 33) there were no AEs, and in the other 2 the occurrence of AEs was unrelated to the exercise intervention. Uncertainty concerning safety is a significant hurdle in the clinical prescription of exercise, especially in the unsupervised model (53).

In a recent study, researchers investigated the safety profile of exercise training in people with MS. The results showed that exercise training was associated with a lower rate of reported relapses than the non-exercise control condition. Specifically, the relapse rate was about 27% lower in the exercise training group (54). In addition, the trial found minimal exercise-related AEs, and no serious AEs were reported in the trials analysed. Although the evidence is limited, these results suggest that exercise training may have a disease-modifying effect on MS (55). However, it is important to note that this speculation is based on minimal evidence, and further research is needed to confirm this hypothesis. It is worth noting that exercise training is considered a form of rehabilitation for people with MS. Rehabilitation plays a crucial role in minimizing the impact of the disease on the lives of people with MS (56–58). Therefore, understanding the safety profile of exercise training is important to ensure that people with MS can engage in physical activity without experiencing AEs or any exacerbation of their symptoms.

### Limitations

In conducting a thorough review of the existing literature, it is important to recognize that some relevant studies may have been inadvertently excluded despite efforts to be comprehensive. We used the 2 largest resource databases for this review, but acknowledge this as a limitation, as the inclusion of additional databases might have provided a more comprehensive analysis. In addition, although scientific articles were included in the systematic review, it is important to recognize that there may be a bias in the published literature, as some articles may not report negative findings. This potential bias could affect the overall conclusions of the review.

One of the main limitations observed in most of the included studies was the considerable variability in the methods used for the exercise interventions and the assessment of outcomes. This variability makes it difficult to synthesize the results and draw definitive conclusions. Therefore, it is imperative that future research establish standardized guidelines and recommendations for assessing outcomes in exercise-based interventions. There was also heterogeneity in the definition of telerehabilitation used in home-based exercise interventions. In addition, the diversity observed in the characteristics of the studies, such as the age and type of MS in the participant population, and the variations in exercise prescription, may have contributed to a reduction in the overall quality of the generalizability of the findings. The heterogeneity of these factors makes it difficult to apply the results universally and highlights the need for more standardized approaches in future studies.

Finally, it is important to recognize that the participants included in trials may not be fully representative of the general population with MS. This potential limitation arises from the selection process, which may have favoured younger or more motivated individuals who preferred technology-based rehabilitation. In addition, many participants may not have had the opportunity to express their exercise preferences. This selection bias could affect the generalizability of the findings and should be taken into account when interpreting the results.

### Future perspectives

Telerehabilitation may lead to increased attractiveness and usefulness of traditionally delivered MS rehabilitation. However, the acceptability and usefulness of the telerehabilitation approach may be challenged by several factors (Table IV).

**Table IV:** Advantages and potential challenges of telerehabilitation in multiple sclerosis

Advantages	Challenges
Autonomy in exercise planning	Ability to progress/achieve exercise goals
Reduced time and travel constraints	Consequences of unsupervised exercise
Self-guided daily physical activities	Limited face-to-face interaction
Mobile health motivational platforms	Exercise data integration in medical record
Low cost	Digital literacy
Increased privacy	Absence of published guidelines

Future development is needed to inform and guide healthcare professionals, researchers, and policymakers regarding the safety of exercise training in people with MS. At the same time, it will be important to determine, where possible, the effect of exercise type, exercise delivery style, participant disability level, and exercise guidelines on exercise safety.

This systematic review showed the effectiveness of home-based telerehabilitation exercises in improving movement skills, including walking, muscle strength, and motor skills. The results showed that the improvement in movement skills was significantly greater in the telerehabilitation exercise group than in the control group. However, further research is necessary to investigate practical approaches to prescribing the intensity of home-based exercises. To improve the effectiveness of home-based telerehabilitation exercises, it would be beneficial to develop and use mobile applications that include features such as monitoring exercise intensity, motivating patients to adhere to the exercise programme, and ensuring correct posture during exercise. In ongoing research (59, 60), these technological advances may help to optimize the outcomes of telerehabilitation interventions.

It is also necessary to focus on the needs of patients and providers of telerehabilitation and the system of reimbursement for this type of rehabilitation by the insurance companies. Further research may lead to high-quality care that benefits patients, carers, and healthcare providers (17). It is important to remember that home exercise programmes cannot be one-size-fits-all and must be tailored to the specific needs and abilities of people with MS. Future research should address the individual problems faced by people with MS, such as fatigue, balance disorders, and muscle weakness.

### Conclusion

Although evidence suggests that telerehabilitation could be a feasible and effective rehabilitation option for people with MS with a beneficial effect on motor function, further research is still needed to determine its effectiveness compared with usual care. The current literature suggests that telerehabilitation is safe and has the potential to provide comparable results in improving movement status and to offer patients another form of rehabilitation that may be appropriate for them. Adherence to telerehabilitation also appears to be similar to regular centre-based rehabilitation. However, more high-quality studies are needed to fully evaluate the effectiveness and efficiency of telerehabilitation in managing the care of people with MS.
